# Early detection of enamel demineralization by optical coherence tomography

**DOI:** 10.1038/s41598-019-53567-7

**Published:** 2019-11-20

**Authors:** Meng-Tsan Tsai, Yen-Li Wang, Ting-Wei Yeh, Hsiang-Chieh Lee, Wen-Ju Chen, Jia-Ling Ke, Ya-Ju Lee

**Affiliations:** 1 Department of Electrical Engineering, Chang Gung University, Taoyuan, 33302 Taiwan; 2 Department of Neurosurgery, Chang Gung Memorial Hospital, Linkou, 33305 Taiwan; 3 Department of Periodontics, Chang Gung Memorial Hospital, Taoyuan, 33378 Taiwan; 4 College of Medicine, Chang Gung University, Taoyuan, 33302 Taiwan; 5 Institute of Electro-Optical Science and Technology, National Taiwan Normal University, Taipei, 11677 Taiwan; 6 Graduate Institute of Photonics and Optoelectronics, National Taiwan University, Taipei, 10617 Taiwan; 7 Department of Electrical Engineering, National Taiwan University, Taipei, 10617 Taiwan

**Keywords:** Optical spectroscopy, Optical techniques

## Abstract

Enamel is the outermost layer of the tooth that protects it from invasion. In general, an acidic environment accelerates tooth demineralization, leading to the formation of cavities. Scanning electron microscopy (SEM) is conventionally used as an *in vitro* tool for the observation of tooth morphology changes with acid attacks. Yet, SEM has intrinsic limitations for the potential application of *in vivo* detection in the early demineralization process. In this study, a high-resolution optical coherence tomography (OCT) system with the axial and transverse resolutions of 2.0 and 2.7 μm in teeth has been utilized for characterizing the effect of the acidic environment (simulated by phosphoric acid) on the enamel topology. The scattering coefficient and the surface roughness of enamel can be directly derived from the OCT results, enabling a quantitative evaluation of the topology changes with demineralization. The dynamic process induced by the acid application is also recorded and analyzed with OCT, depicting the evolution of the demineralization process on enamel. Notably, the estimated enamel scattering coefficient and surface roughness significantly increase with the application time of acid and the results illustrate that the values of both parameters after demineralization are significantly larger than those obtained before the demineralization, illustrating both parameters could be effective to differentiate the healthy and demineralized teeth and determine the severity. The obtained results unambiguously illustrate that demineralization of the tooth surface can be successfully detected by OCT and further used as an indicator of early-stage cavity formation.

## Introduction

Tooth is a hard structure that can cut and crush food before swallowing and digesting. Human teeth are divided into four types including incisors, canines, premolars, and molars, which perform different functions during food chewing^1^. Moreover, the tooth body contains three parts: a crown, a neck, and a root^2^. The crown is the outmost part of the tooth structure not surrounded by the soft tissue that is located above the cementoenamel junction and below the crown; and the neck forms a line between the crown and the root, which is fastened in the jawbone and acts as a tooth support. The tooth anatomy includes enamel, dentin pulp, and cementum^3^. The enamel is the hardest part of the tooth and primarily contains hydroxyapatite (crystalline calcium phosphate)^4^. The reaction of sucrose in food with bacteria on the crown surface produces acid that causes calcium loss from the enamel layer of the crown^5^. In contrast, saliva acts as a reservoir of minerals that inhibits the demineralization of the enamel layer^6^. Normally, the rates of demineralization and remineralization on the tooth surface are balanced to keep the teeth healthy. However, when teeth are exposed to an acidic environment for a long period, it easily produces an unbalance between the demineralization and remineralization processes^7^. Therefore, in the beginning of dental caries, demineralization occurs on the tooth surface due to acid exposure that gradually destroys the enamel structure. As a result, dental caries causes tooth breakdown because of the presence of acid-induced bacteria in the oral cavity that dissolves the tooth structure. In the past, X-ray analysis has become a golden standard for the diagnosis of dental caries; however, it is difficult to diagnose its early stage or tooth demineralization using this method^8^.

Optical coherence tomography (OCT) is a label-free cross-sectional imaging technique that is able to provide depth-resolved information about biological samples^9^. Similar to ultrasound imaging, OCT utilizes an interferometer to acquire a depth-dependent backscattered signal from the sample to retrieve its depth-resolved structure^10^. Previous studies have successfully demonstrated that OCT may be used for various biomedical applications, such as, ophthalmology^11,12^, dermatology^13,14^, cardiology^15,16^, and tooth evaluation^17,18^. It was found that OCT could serve as a potential diagnostic tool for caries, cracks, and tooth defects^19–22^. Moreover, some groups proposed to use OCT for the detection of the demineralization process, which was considered an early sign of caries. Popescu *et al*. estimated attenuation coefficients from the OCT depth profiles of demineralized and remineralized teeth^23^. They found that the enamel reflectivity increased with demineralization, but decreased with remineralization. Zhou *et al*. used OCT for observing teeth demineralization; the obtained data strongly correlated with the images acquired by confocal laser scanning microscopy^24^. Additionally, Alghilan *et al*. applied cross-polarization OCT to determine the influence of demineralization on the tooth surface roughness and severity^25^. Chew *et al*. used quantitative light-induced fluorescence and OCT for observing the progression of enamel demineralization, and evaluated the changes in the surface microhardness, fluorescence loss, and the OCT backscattered intensity by comparing the results obtained at the baseline time before and after the erosion^26^. Aden *et al*. demonstrated the utilization of 3D OCT images to detect the early-stage erosion based on the measurement of time-dependent correlation^27^. Later, Aden *et al*. also measured the induced changes in optical properties such as the backscattered intensity from time-series 3D-OCT imaging of bovine enamel with different demineralization solutions^28^. Austin *et al*. proposed an analysis method for evaluating the influence on the OCT backscattered intensity of enamel surface due to orange juice rinsing^29^. Habib *et al*. investigated the surface roughness changes of the eroded dentin based on the estimation of the attenuation coefficient from the OCT backscattered intensity^30^. The previous reports indicated that the teeth demineralization causing the changes in surface morphology and optical properties of enamel which can be identified by OCT. However, the dynamic progress of demineralization is rarely discussed, and it might be a problem for evaluation of demineralization with the previously proposed methods for clinical practice since it is difficult to repeatedly scan the same tooth location. Therefore, a higher axial or transverse resolution would be a better solution for providing more accurate information of optical properties and surface structure of enamel.

In this study, a high-resolution OCT system was used for the real-time observation of the tooth demineralization process and related changes in the micromorphology and optical scattering on the tooth surface. To induce tooth demineralization and simulate the acidic environment in the oral cavity, 37% phosphoric acid gel was applied to the enamel, whose scattering coefficient and surface roughness were determined before and after demineralization to quantify the observed influence on the enamel scattering characteristics and morphological changes on its surface, respectively.

## Methods

### Experimental setup

**Figure 1:**
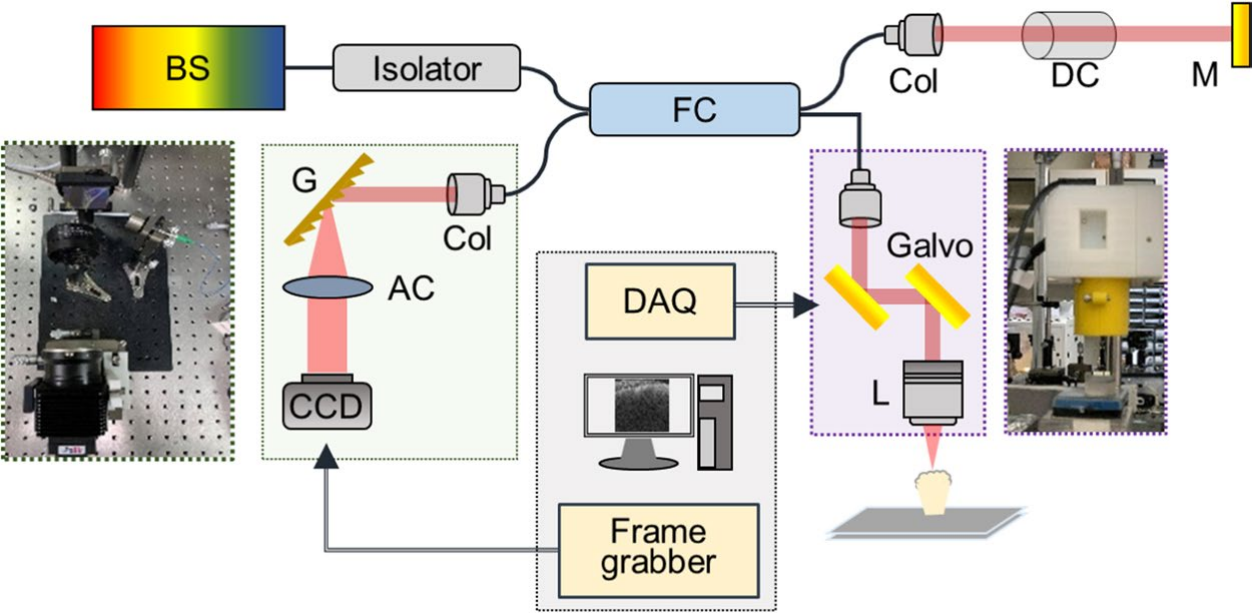
Schematic diagram of a high-resolution SD-OCT system used for teeth scanning and photos of the spectrometer and the scanning end. BS: broadband source, FC: fiber coupler, Col: collimator, M: mirror, DC: dispersion compensator, Galvo: galvanometer, G: diffraction grating, AC: achromatic lens, and CCD: linescan camera.

To characterize the changes on the tooth surface due to demineralization, a high-resolution spectral-domain OCT (SD-OCT) system was developed for teeth scanning (see Fig. 1). The utilized broadband light source contained a superluminescence diode with a full-width at half maximum of 150 nm with a center wavelength of 850 nm. An isolator was connected to the output of broadband light source to prevent from the back reflection and then, a fiber-based Michelson interferometer was attached to the output of the isolator. A two-axis galvanometer (GVS002, Thorlabs, New Jersey, USA) was utilized for lateral scanning, and a scanning lens (LSM02-BB, Thorlabs, New Jersey, United States) was used to focus the optical beam on the sample. The dispersion induced by the scanning lens was compensated by a dispersion compensator. Finally, the interference signal was detected by a spectrometer consisting of a beam expander, a diffraction grating (GR50-1208, Thorlabs, New Jersey, USA), an achromatic lens (AC508-150-B-ML, Thorlabs, New Jersey, USA), and a linescan camera (spL4096-140km, Basler, Ahrensburg, Germany). The photos of the spectrometer and the scanning end are also shown in Fig. 1. To synchronize the lateral scanning and frame acquisition processes, a DAQ board (PCIe-6229, National Instruments, Austin, USA) with multichannel analog outputs was used. The line rate of the line scan camera was set to 100 kHz corresponding to a system frame rate of 100 Hz. The measured axial and transverse resolutions were equal to 3 and 4 μm in air, respectively. The volume data obtained by three-dimensional (3D) OCT imaging consisted of 500 B-scans containing 1000 A-scans each

### Sample preparation and application procedure

In this study, healthy human teeth diagnosed by clinical dental physicians were used. Human extracted teeth were collected from the Taoyuan branch of the Chang Gung Memorial Hospital (Taiwan) after obtaining a written informed consent. The utilized experimental procedure was approved by the Gung Medical Foundation Institutional Review Board (No. 201700937b0). In this study, all experimental methods were performed in accordance with the relevant guidelines and regulations approved by the Gung Medical Foundation Institutional Review Board (No. 201700937b0). To investigate the effects produced by the acid environment on the tooth surface, 37% phosphoric acid gel (AM-TOUCH DENTAL, Valencia, CA) was applied to accelerate the its demineralization process. Before the acid application, the tooth was thoroughly cleaned with a toothbrush and an ultrasonic cleaner and then fixed with clay in an upright position. Subsequently, a fixed amount of the gel (3 mL) was applied onto the crown surface for either 120 or 300 s. After the application, the gel was removed from the tooth surface, which was cleaned again by the toothbrush and ultrasound cleaner to eliminate possible traces of various chemical products. To investigate the dynamic change of the demineralization process, the tooth was repeatedly scanned by OCT at different time points before, during, and after the acid application. Moreover, to validate the obtained OCT results, the OCT scanning area was subsequently examined by scanning electron microscopy (SEM).

### Data analysis

To quantify the influence of the acidic environment on the scattering properties of the enamel, its scattering coefficient *μ*_*s*_ was estimated. In a previous work, the measured scattering coefficient of the tooth enamel ranged from 1.5 to 10.5 mm^−1^ at a wavelength between 543 and 1060 nm, indicating that the latter represented a tissue with relatively weak scattering properties^31,32^. For such a tissue, the value of *μ*_*s*_ can be determined by a single scattering model combined with dynamic focusing. To accurately simulate the dynamic focusing behavior of an OCT system, a confocal point spread function *PCF(z)* was introduced:

1 $$PCF(z)=1/[1+{(\frac{z-{z}_{cf}}{{z}_{R}})}^{2}]$$

where *z* is the depth range, while *z*_*cf*_ and *z*_*R*_ are the depth location of the focal plane and Rayleigh length, respectively. Therefore, the OCT depth profile *I(z)* can be written as

2 $$I(z)\alpha \sqrt{\frac{{e}^{-2{\mu }_{s}z}}{[1+{(\frac{z-{z}_{cf}}{{z}_{R}})}^{2}]}}$$

Here, the factor 2 reflects the one-round trip of the light beam since the OCT system receives a backscattered signal from the tissue. Moreover, the OCT signal in the tissue exhibits an exponential decay according to Beer-Lambert law. *I(z)* can be obtained through the Fourier transformation of the interference spectrum from the OCT system; ${z}_{cf}$ was approximately set to 200 μm beneath the tooth surface; and the ${z}_{R}$ of the utilized OCT system was equal to about 90 μm. Since demineralization may influence the roughness of the tooth surface, its magnitude was estimated from the OCT results. As compared to the inner tooth structure, the tooth surface exhibits a higher backscattered intensity due to the large difference between the refractive indices of air and the enamel. Therefore, a threshold value can be established to reject the weaker backscattered signal from the inner structure of the tooth and obtain a digitized OCT image. Using this image, the enamel surface layer can be characterized to determine its top and bottom locations (${z}_{t}$ and ${z}_{b}$) for each A-scan. In this case, the root mean square of the surface roughness can be estimated as follows:

3 $${R}_{q}=\sqrt{\frac{1}{n}{\sum ({z}_{b}-{z}_{t})}^{2}}$$

where *n* is the total number of A-scans for estimation of surface roughness.

## Results

**Figure 2:**
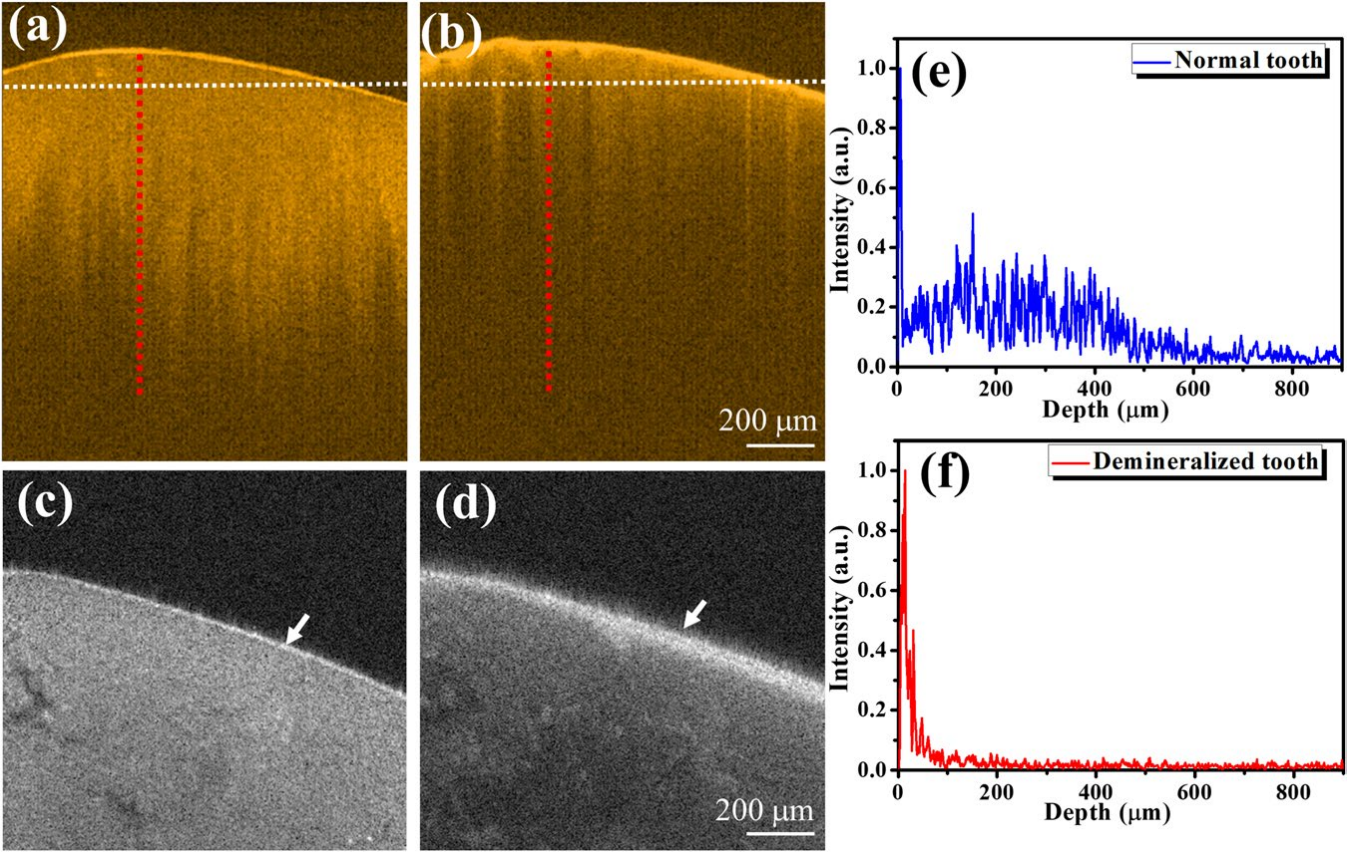
2D OCT images of the tooth obtained (**a**) before and (**b**) after the acid application for 120 s. Corresponding *en-face* images of the same tooth extracted from the 3D OCT data and obtained (**c**) before and (**d**) after the acid application for 120 s, respectively. The white lines indicate the depths of the *en-face* images. Panels (**e**,**f**) contain the average OCT depth profiles obtained at the locations indicated by the red lines in panels (**a**,**b**), respectively.

To examine the difference between the healthy and demineralized teeth, the tooth surface was scanned before the acid application for 120 s, and a volume dataset with a size of 1.2 × 1.2 × 1 mm^3^ was recorded. Subsequently, the phosphoric acid gel was applied for 120 s to induce tooth demineralization and then removed from the tooth surface. The demineralized tooth was cleaned with the brush and ultrasound cleaner, and the same tooth area was scanned by OCT. Figure 2(a,b) show the two-dimensional (2D) OCT images obtained at the same location before and after the acid application, respectively. Moreover, the corresponding *en-face* OCT images extracted from the 3D images of Fig. 2(a,b) are shown in Fig. 2(c,d), respectively. The obtained results indicate that the backscattered intensity around the tooth surface became stronger, while the thickness of the surface layer increased after the acid application. It was previously shown that the acid-induced demineralization caused an appearance of enamel rods on the etched enamel surface^33^. On the other hand, the enhanced backscattered intensity is observed on the tooth surface in Fig. 2(b,d), which is likely related to the presence of these rods. Figure 2(e,f) show the averaged data obtained for 10 neighboring A-scans as indicated by the red lines in Fig. 2(a,b), respectively. The A-scans also demonstrate that the backscattered intensity gradually decreases with the depth of the normal tooth, but its magnitude determined for the tooth surface is much higher than that measured for the deep region after demineralization. These results are in good agreement with the data obtained in a previous study, indicating that the attenuation coefficient of the demineralized teeth is larger than that of the normal ones^23^. Hence, the higher attenuation coefficients of the demineralized teeth were caused by the stronger scattering on their surface, which limited the optical penetration into the tooth body.

**Figure 3:**
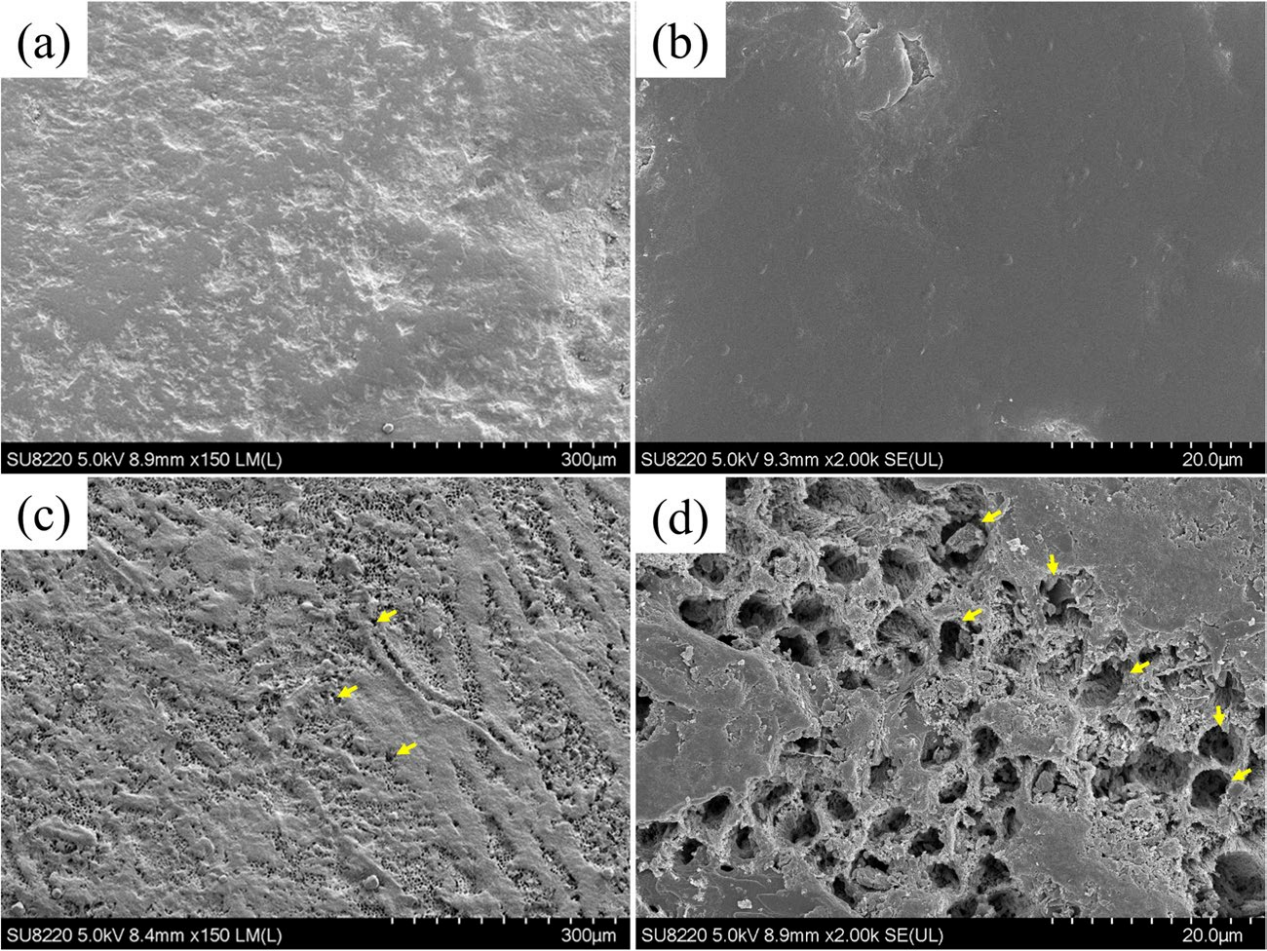
SEM images of the normal and demineralized teeth obtained before the acid application at (**a**) 150× and (**b**) 2000× magnifications and after the acid application at (**c**) 150× and (**d**) 2000× magnifications. The yellow arrows indicate the appearance of enamel rods after etching the enamel surface.

To validate the OCT results presented in Fig. 2, the differences between the healthy and demineralized teeth were examined by SEM. Figure 3(a,b) show the SEM images of the healthy tooth obtained at 150× and 2000× magnifications, respectively. In contrast, Fig. 3(c,d) display the SEM images of the demineralized tooth obtained at the same magnifications. In contrast to the results presented in Fig. 3(a,b), the enamel surface after etching contains the hexagonal structures denoted by the yellow arrows in Fig. 3(c,d). These structures represent enamel rods or prisms consisting of hexagonal hydroxyapatite crystals^34^. Therefore, the SEM images revealed that teeth demineralization caused the dissolution of superficial calcium hydroxyapatite, which exposed the enamel rods and increased the backscattered intensity from the tooth surface as shown in Fig. 2(b,d).

**Figure 4:**
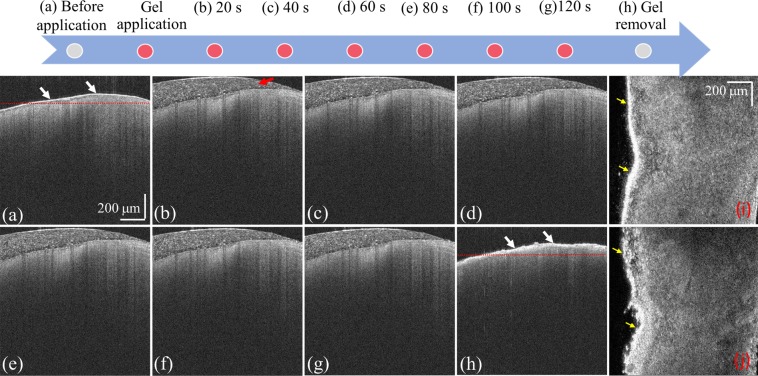
Time-series OCT images of the same healthy tooth obtained (**a**) before the application of the acid gel; after the gel application for (**b**) 20, (**c**) 40, (**d**) 60, (**e**) 80, (**f**) 100, and (**g**) 120 s; and (**h**) after the gel removal. Panels (**I**,**j**) contain the *en-face* images extracted from the 3D images depicted in panels (**a**,**h**), respectively.

To further investigate the dynamics of the demineralization process, OCT scans were obtained for one healthy tooth at various time intervals (Fig. 4). Before the application of the acid gel onto the tooth surface, the tooth was scanned by OCT to acquire a 3D dataset (Fig. 4(a)). In this image, the strong and smooth backscattered distribution on the tooth surface is indicated by the white arrows. After that, the acid gel was applied onto the tooth enamel, and the same area was repeatedly scanned by OCT after 20, 40, 60, 80, 100, and 120 s as shown in Fig. 4(b–g) (the red arrow indicates the applied gel on the tooth surface). After 120 s, the gel was removed; the tooth was cleaned by the toothbrush and ultrasound cleaner; and the same area was scanned again as shown in Fig. 4(h). Compared with Fig. 4(a,h) shows that the backscattered intensity from the enamel surface became stronger and that the corresponding depth range decreased. Moreover, the OCT intensity signal rapidly decreased, further limiting the OCT penetration depth. The corresponding *en-face* images of Fig. 4(a,h) with depths denoted by the red lines are displayed in Fig. 4(j), respectively. They show that the roughness of the tooth surface significantly changed after demineralization.

**Figure 5:**
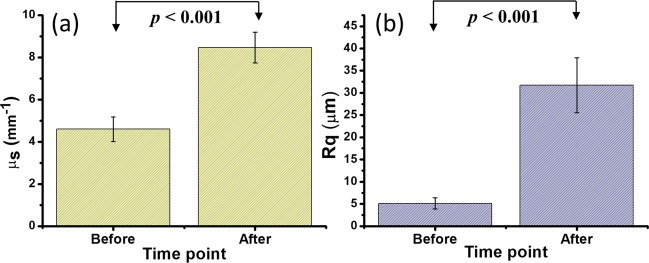
(**a**) Tooth scattering coefficients and (**b**) surface roughness values estimated before and after the demineralization for 120 s. The error bars represent the estimated standard deviations of *μ*_*s*_ and *R*_*q*_.

It was found that demineralization induced the morphological change of the enamel surface, which increased the backscattered intensity from the tooth. Therefore, healthy teeth were used to statistically evaluate the observed changes in the scattering coefficient and surface roughness caused by demineralization. Before the application of the acid gel onto the enamel surface, the healthy teeth were scanned by OCT to acquire 3D images, after which phosphoric acid was applied onto the enamel surface for 120 s. Subsequently, the gel was removed, and the teeth were cleaned by the toothbrush and ultrasound cleaner. Finally, the same teeth areas were scanned again by OCT. Since each 3D OCT dataset obtained for one tooth sample consists of 500 B-scans containing 1000 A-scans each, the average scattering coefficient of each tooth can be estimated by considering the 400−600th A-scans of every B-scan in the 3D OCT dataset (a similar procedure was used for determining the average surface roughness of each tooth). Figure 5 shows the scattering coefficients and surface roughness values obtained for the twenty tooth samples before and after demineralization. In Fig. 5(a), the mean scattering coefficient before demineralization was 4.60 mm^−1^; however, its value increased to 8.46 mm^−1^ after the demineralization for 120 s. Similarly, the measured *R*_*q*_ magnitude also increased from 5.11 μm to 31.7 μm after demineralization. The obtained scattering coefficients and surface roughness values were statistically significant according to Student’s t-test (*p* < 0.001).

**Figure 6:**
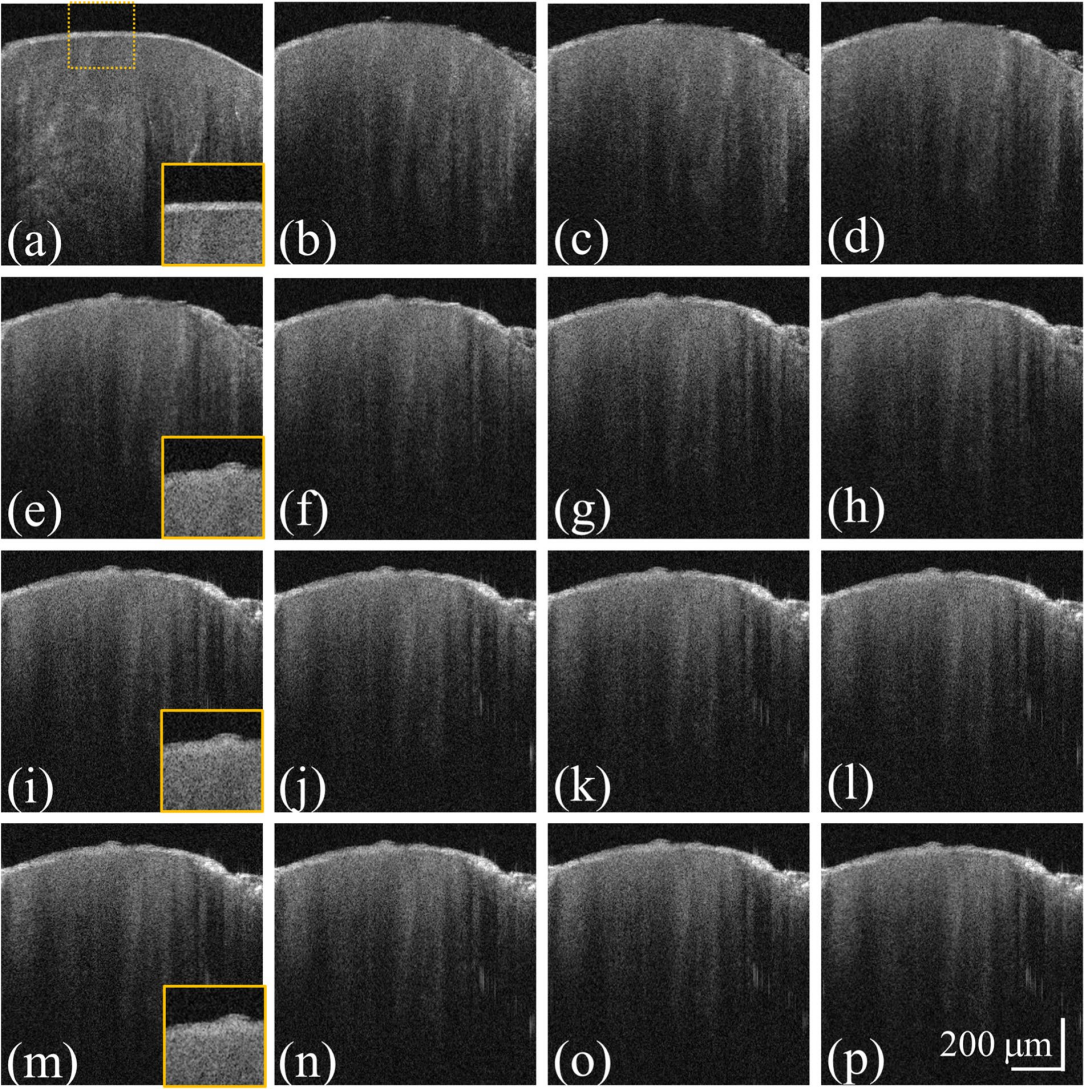
Time-series OCT images of the healthy tooth obtained (**a**) before and after demineralization for (**b**) 20, (**c**) 40, (**d**) 60, (**e**) 80, (**f**) 100, (**g**) 120, (**h**) 140, (**i**) 160, (**j**) 180, (**k**) 200, (**l)** 220, (**m**) 240, (**n**) 260, (**o**) 280, and (**p**) 300 s. The magnified regions are indicated by the yellow-dash squares in the bottom right corners of panels (**a**,**e**,**I,m**).

In Fig. 4, the acid gel was applied onto the tooth surface, which was continuously scanned by OCT. However, it was difficult to directly determine the influence of demineralization on the tooth scattering coefficient and surface roughness. Therefore, to investigate the effect of the application period on the enamel properties, the following procedure was repeated every 10 s until a total demineralization time of 300 s was reached. In this experiment, the gel was applied onto the surface of a healthy tooth for 10 s followed by its removal and cleaning with the toothbrush and ultrasound cleaner. After that, the treated tooth area was scanned by OCT. Figure 6 shows the time-series OCT images of the healthy tooth obtained at various time points of the demineralization process. Figure 6(a) acquired before demineralization shows the existence of a smooth and clear surface boundary between air and the enamel surface. The OCT images obtained after the acid application for 20, 40, 60, 80, 100, 120, 140, 160, 180, 200, 220, 240, 260, 280, and 300 s are displayed in Fig. 6(b)−(p). They show that the enamel surface became rougher at larger demineralization times. Additionally, the magnified regions indicated by the yellow-dash squares and depicted in the bottom right corners of Fig. 6(a,e,i,m) reveal that the enamel surface became gradually blurred and unclear due to demineralization.

**Figure 7:**
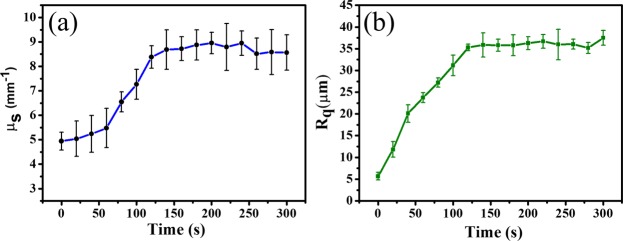
(**a**) Scattering coefficients and (**b**) surface roughness values estimated for the healthy tooth (**a**) before and after demineralization for (**b**) 20, (**c**) 40, (**d**) 60, (e) 80, (**f**) 100, (**g**) 120, (**h**) 140, (**i**) 160, (**j**) 180, (**k**) 200, (**l**) 220, (**m**) 240, (**n**) 260, (**o**) 280, and (**p**) 300 s. The error bars represent the estimated standard deviations of *μ*_*s*_ and *R*_*q*_.

To quantitatively evaluate the influences of demineralization on the tooth scattering coefficient and surface roughness, the data presented in Fig. 6 was processed using Eqs (2) and (3). The obtained relationships between the scattering coefficient/surface roughness and the application period are depicted in Fig. 7. Figure 7(a) shows that the estimated scattering coefficient increased with the application time. The obtained values of the surface roughness exhibited the same trend (Fig. 7(b)): its magnitude gradually increased after the acid application and leveled out at application times greater than 120 s.

## Discussion

The results of this study demonstrated the possibility of using OCT for the early detection of tooth demineralization, which could be an important sign of early-stage caries. Because the presence of an acid in the oral cavity causes the calcium loss from the enamel, it significantly affects its surface and gradually destroys the tooth structure to ultimately form dental cavities. Previously, enamel rods (prisms) with diameters of 3–6 μm appeared after etching the enamel surface with acid^34^. Such rods are composed of hexagonal hydroxyapatite crystals with lengths of approximately 65 nm and widths of 30 nm, which move from the enamel-dentin interface to the enamel surface. However, it is difficult to directly observe the effect of acidification on enamel rods or hexagonal hydroxyapatite crystals. Therefore, to examine the influence of etching on the enamel layer or enamel rods, high-resolution OCT was used in this work. The obtained SEM images demonstrated that the enamel surface of the healthy teeth was smooth and that the structures beneath it were fully covered. In contrast, when the enamel surface was etched, the enamel rods were exposed to air or acid. Thus, the OCT backscattered intensity from the enamel surface increased after demineralization due to the scattering enhancement by the uncovered enamel rods or hexagonal hydroxyapatite crystals. To quantify the produced changes on the enamel surface, its scattering coefficient was estimated. The mean scattering coefficient obtained for the normal teeth was 4.60 mm^−1^, while the value determined for the demineralized teeth was 8.46 mm^−1^. This increase in the enamel scattering coefficient was partially caused by the scattering enhancement by the uncovered enamel rods or hexagonal hydroxyapatite crystals. The enamel scattering coefficient measured in a previous study was 1.5 mm^−1^ at 1053 nm^31^, which slightly differed from the data reported in this work likely due to the different measurement approaches and the difference in wavelength. In particular, a depth range of 800 μm from the enamel surface was utilized during each OCT A-scan for the estimation of the scattering coefficient in this study, and an exponential decay was used for curve fitting according to Eq. (2). However, the selected depth range may slightly influence the fitting results, leading to a different value of the scattering coefficient. Using the proposed method, the estimated scattering coefficients of the healthy and demineralized teeth can be effectively differentiated. Because the enamel surface can be etched by acid, the distribution of the OCT signal intensities scattered from the tooth surface was obtained to estimate the enamel surface roughness. Although the proposed method can effectively identify changes in the tooth surface morphology during demineralization, it may produce estimation errors with respect to the more accurate values determined from SEM images. Moreover, the obtained OCT results revealed the absence of significant variations of the scattering coefficient and surface roughness after the demineralization for more than 120 s because of the scattering enhancement of the tooth surface that further limited the optical penetration into the tooth body after the specified period. Currently, the design of the scanning end is similar to our previous setup used for skin scanning, being able to be used as a microscopic or a handheld type^35^. However, the current probe design in this study is too bulky, making clinical applications difficult. Additionally, we also demonstrated a handheld probe for *in vivo* scanning various types of mucosae in the oral cavity which was introduced in our previous report^36^. In this study, a high-resolution OCT was developed for investigation of demineralization process of enamel with the axial and transverse resolutions of 2.0 and 2.7 μm in teeth. Integration of high-resolution OCT and the handheld probe would be able to apply the developed method in this study for *in vivo* detection of the early-stage demineralization in the future.

Demineralization can be considered an early sign of tooth caries due to the excessive reaction induced by an acid. Therefore, the identification of tooth demineralization plays an important role in the detection of early-stage caries. In this study, high-resolution OCT was used for investigating the morphological and scattering changes of the tooth enamel surface due to demineralization. To simulate the acid environment in the oral cavity, 37% phosphoric acid gel was applied onto the enamel. Additionally, the effects of demineralization on the tooth surface were quantitatively evaluated to estimate the enamel scattering coefficient and surface roughness. It was found that the scattering coefficient increased after demineralization because of the dissolution of calcium hydroxyapatite in the enamel structure, which also produced surface irregularities. Finally, the dependence of the demineralization process on the application time was investigated. The observed changes in the enamel surface morphology and scattering coefficient became insignificant when the application period exceeded 120 s. Thus, tooth demineralization can be effectively studied by high-resolution OCT to facilitate early detection of tooth caries.
